# Titanium(IV)-induced cristobalite formation in titanosilicates and its potential impact on catalysis

**DOI:** 10.1007/s10853-018-2869-0

**Published:** 2018-09-06

**Authors:** Ayomi S. Perera, Jeremy K. Cockcroft, Panagiotis Trogadas, Haiyue Yu, Nidhi Kapil, Marc-Olivier Coppens

**Affiliations:** 1 Department of Chemical Engineering, Centre for Nature Inspired Engineering, University College London, Torrington Place, London, WC1E 7JE UK; 2 Department of Chemistry, University College London, 20 Gordon Street, London, WC1H 0AJ UK

## Abstract

Cristobalite, a crystalline form of silica, is shown to be formed within an amorphous titanosilicate, at previously unknown conditions. Mesoporous titanosilicate microspheres (MTSM) were synthesized as efficient catalysts for the epoxidation of cyclohexene with *tert*-butyl hydroperoxide. High-resolution transmission electron microscopy revealed the presence of crystals in this predominantly amorphous material, after calcination at 750 °C. When calcined at 800 °C, the crystals were identified via PXRD as predominantly cristobalite, which possibly marks its first observation in titanosilicates at such a low temperature, without adding any alkali metals during synthesis. Catalytic experiments conducted with MTSM materials calcined at temperatures varying from 650 to 950 °C, reveal that the amount of cristobalite formed increases with temperature, and that it has a significant impact on the pore structure, and, remarkably, correlates with the catalytic activity of titanosilicates.

**Electronic supplementary material:**

The online version of this article (10.1007/s10853-018-2869-0) contains supplementary material, which is available to authorized users.

## Introduction

Titanosilicates are versatile materials with applications in catalysis, ion-exchange and membrane technology in petrochemical, pharmaceutical, food and water purification industries [1, 2]. In particular, they are commercially valuable as highly selective heterogeneous catalysts for oxidation of alkenes, to form industrially relevant organic epoxides and alcohols, which are precursors for various agrochemicals, food preservatives and pesticides [3–5]. Titanosilicates hold significant advantages over other conventional catalysts in these processes, as, in addition to product selectivity, they can also be used under mild reaction conditions, to form environmentally benign byproducts, are hydrothermally stable, and generate high product yields and substrate conversions [6–9]. Their synthesis can also be tailored to form micro-, meso- or hierarchical micro-mesoporous and layered structures, for the catalysis of a wide range of organic substrates [2, 6, 10–12].

The customization of the physical structure of titanosilicates can be achieved via temperature-dependent phase transformations, in order to generate various frameworks, and layered structures, that target specific catalytic reactions or ion-exchange applications [13–16]. Calcination is often used in such processes, to burn off templating agents and other impurities [17]. In addition, with increasing calcination temperature, titanosilicates transform between various structural phases, such as amorphous-to-crystalline-to-layered [14] or crystalline-to-amorphous-to-layered [13]. Understanding the chemical and physical conditions of such transformations is a key factor in the effective synthesis of titanosilicates.

Phase transitions are usually observed when ionic forms of alkali metals, such as Na and K, are incorporated during the synthesis of titanosilicates [13–16, 18]. In silica materials, it is well known that the presence of Group 1 elements (such as Na and Li) facilitate phase separations by significantly lowering the transition temperature [19] and similar effects are seen in titanosilicates. In addition, the presence of crystalline anatase TiO_2_ phases commonly contributes to the phase heterogeneity of titanosilicates [17, 20]. Increasing the Ti content during synthesis of titanosilicates has been shown to increase the formation of anatase, a crystalline form of TiO_2_ [21]. It has long been established that the catalytic activity of titanosilicates is brought about by the isolated tetrahedral Ti^4+^ sites within the silica matrix; [22–24] thus, the presence of catalytically inactive TiO_2_ phases is highly undesirable. Furthermore, TiO_2_ causes the decomposition of oxidants used in titanosilicate-catalyzed reactions [25], adding to further catalytic inefficiency. Therefore, minimizing the formation of crystalline TiO_2_ phases is crucial in developing effective titanosilicate catalysts. What is less understood, however, is whether the presence of crystalline silica phases, such as cristobalite, has any effect on the catalytic activity of titanosilicates. This is partly due to the fact that such phases are present in small amounts and are not often identified.

Cristobalite is a crystalline form of silica, which is naturally found in volcanic rocks [26–28]. Pure silica gels can form cristobalite by phase transformation at temperatures as high as ~ 1470–1705 °C [29–31]. However, with the presence of alkali metals such as Na and Li (usually found in volcanic rocks), this temperature can be reduced to as low as 900 °C [19, 32]. Reports of cristobalite formation in titanosilicates are rare, and when reported, cristobalite was present in small quantities and was found only when sources of Na were included in stoichiometric amounts during the synthesis of titanosilicates [33–35]. There is no evidence in the literature indicating that the presence of Ti^4+^ itself has any effect on the formation of such crystalline silica phases.

The goals of this study were: (1) to investigate the factors that induce the formation of cristobalite formed in titanosilicates at low temperatures, without the incorporation of other phase transition inducing agents and (2) whether these cristobalite phases have an effect on the catalytic activity of titanosilicates. The results obtained provide evidence of formation of cristobalite at temperatures much lower than those reported previously, without the addition of alkali metals or organic ligands. The impact of the crystalline phases on the catalytic activity of titanosilicates was thoroughly investigated and discussed. These findings may be of critical importance in understanding factors that affect catalytic activity of titanosilicates, as well as provide insight into the importance of controlling calcination conditions in large-scale synthesis.

## Materials and methods

### Synthesis of titanosilicate microspheres

Synthesis was based on previously published procedures [17, 36]. All chemicals were purchased from Sigma-Aldrich and used without further purification.

#### General procedure

1.0 ml of Ti(IV) *n*-butoxide (99%) was added dropwise to 30.0 ml of DI water (18.2 MΩ), at 4 °C, under magnetic stirring, in order to form Ti(OH)_4_ precipitate. The precipitate was filtered under vacuum and washed with DI water. Ti(OH)_4_ was then dissolved in 4.0 ml of 4 N HNO_3_ to produce the active species TiO(NO_3_)_2_. TiO(NO_3_)_2_ was then mixed with a solution of 6.6 ml tetraethyl orthosilicate (TEOS, 98%) and 2.0 ml of ethanol and stirred vigorously for 30 min to form isolated Ti^4+^ sites in the silica matrix. Next, surfactant templating was carried out to create mesoporous microspheres: The above solution was transferred to a mixture of 26.1 g kerosene and 7.9 g Span 80, and homogenized with an Ultra-Turrax homogenizer, at 3000 rpm for 2 h, at 80 °C. The microspheres formed were then vacuum-filtered and washed with acetone and DI water, followed by drying at 80 °C for 2 h. This synthesis procedure was repeated seven times. Finally, the seven samples thus obtained were calcined at different temperatures, namely 650, 700, 750, 800, 850, 900 and 950 °C for 6 h. Hereafter, these samples will be referred to as MTSM-650, MTSM-700, etc.

#### Synthesis of silica microspheres

Silica microspheres were synthesized using the same procedure described in “General procedure” section, without the use of the titanium reagent, hence, 4.0 ml of 4 N HNO_3_ was mixed with a solution of 6.6 ml tetraethyl orthosilicate (TEOS, 98%) and 2.0 ml ethanol and stirred vigorously for 30 min. The microspheres were made mesoporous by surfactant templating, followed by drying, as stated in “General procedure” section. The silica samples thus formed were calcined for 6 h at various temperatures, namely 650, 750, 850 and 950 °C. Hereafter, these will be referred to as S-650, S-750, etc.

### Characterization

HRTEM images were taken with a JEOL 2100 microscope operating at 200 keV. The samples were dispersed in methanol and then dried on a Holey carbon film Cu grid, for TEM measurements. Powder X-ray diffraction (PXRD) patterns were obtained with a Stoe STADI-P diffractometer equipped with a Mythen 1 K detector and using Cu Kα_1_ radiation (40 kV and 30 mA). Samples were ground and filled into 0.7 mm X-ray glass capillaries. Nitrogen adsorption/desorption isotherms were conducted on a Quantachrome Autosorb iQ_2_, using the NLDFT method to evaluate surface area, pore volume and pore size distributions, from the adsorption branch of the isotherms [37]. XPS spectra were recorded on a Thermo Scientific spectrometer with Cu Kα radiation. The analyzer was set at a pass energy of 20 eV for high-resolution spectra of all the individual elements in each sample tested. Approximately 2–5 mg of each powder sample was mounted on a stainless-steel sample holder. The background was determined using the Shirley-type background correction, and the curves were fitted with Gaussian and Lorentzian product functions.

### Catalytic experiments

The MTSM and silica samples were catalytically characterized via epoxidation of cyclohexene with TBHP according to a previously reported procedure [36, 38]. 25 mmol of cyclohexene was mixed with 20 mL of decane as solvent in a batch reactor, followed by 100 mg of MTSM catalyst. Next, approximately 7 g of 4 Å molecular sieves were added to the reactor in order to remove moisture from the mixture. The mixture was stirred at 60 °C for 30 min. The 5.5 M TBHP in decane solution was dried by adding 1 g of molecular sieves to 5 ml of the TBHP solution and storing overnight, prior to the reaction, to remove any adsorbed water. The reaction was initiated by adding 5.5 mmol of the dried TBHP solution. Samples from the reaction mixture were withdrawn at relevant time periods and analyzed by gas chromatography using a GC-2014 Shimadzu Gas Chromatograph, employing a ZB-WAXplus Zebron capillary GC column. Calibration curves for TBHP, cyclohexene and cyclohexene oxide, were obtained using standard solutions, and used to determine the relevant concentrations of the reaction mixtures, via GC analysis.

## Results and discussion

### Formation of cristobalite phases within amorphous titanosilicate catalysts

**Figure 1:**
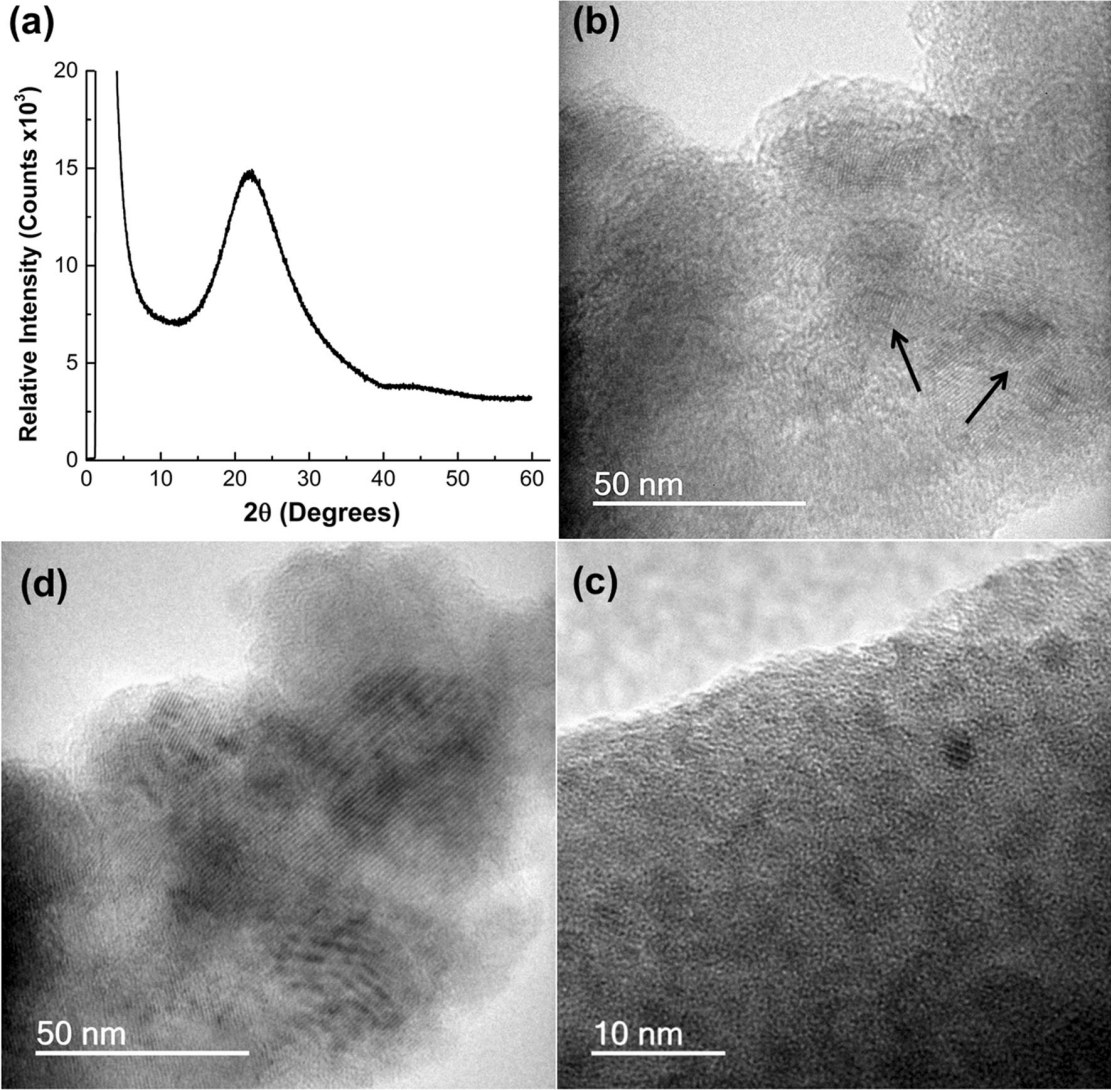
**a** PXRD data of MTSM-750-3, showing one broad peak, indicating that the material is amorphous, **b**, **c** and **d** TEM images of MTSM-750-7, -3, and -2, respectively, showing the presence of crystalline phases, among the predominant amorphous phase

In a previous publication, we reported how the Doehlert matrix statistical approach was used to optimize the synthesis conditions of mesoporous titanosilicate microspheres (MTSM), leading to the development of improved mesoporous catalysts, in a time-effective manner [36]. During this study, two synthetic parameters, i.e., surfactant weight and homogenizing temperature were changed simultaneously, in order to investigate synthesis conditions that would lead to the optimal catalyst (ESI, Table S1). Three MTSM samples were identified as superior catalysts (MTSM-750 samples 7, 3 and 2, in decreasing order of catalytic activity), based on product yield and selectivity obtained during the reaction. These samples were all calcined at 750 °C, in order to remove the surfactant and oil mixture added during the synthesis for the purpose of templating. PXRD measurements performed on these titanosilicate samples revealed that the materials were completely amorphous with no indication of crystalline peaks (Fig. 1a). However, TEM imaging surprisingly indicated that the three most catalytically active samples mentioned above contained small regions of crystalline phases within the predominant amorphous phase (Fig. 1b–d). One sample in particular, MTSM-750-3, showed more crystalline regions compared to any other sample, yet showed no indication of crystallinity in the PXRD within the limits of detection.

The enhanced catalytic activity of the above-mentioned MTSM samples was found to be a result of a combination of factors, such as accessible mesoporosity, high surface area and large pore diameters, together with increased amounts of isolated Ti^4+^ active centers. Samples with high amounts of micropores that can act as bottlenecks to prevent reagent molecules from reaching the Ti^4+^ active sites were found to be less catalytically active [36]. For the current study, experiments were conducted to, first, identify the crystalline phases formed during synthesis of titanosilicates and, secondly, investigate whether these had any impact on their catalytic activity.

**Figure 2:**
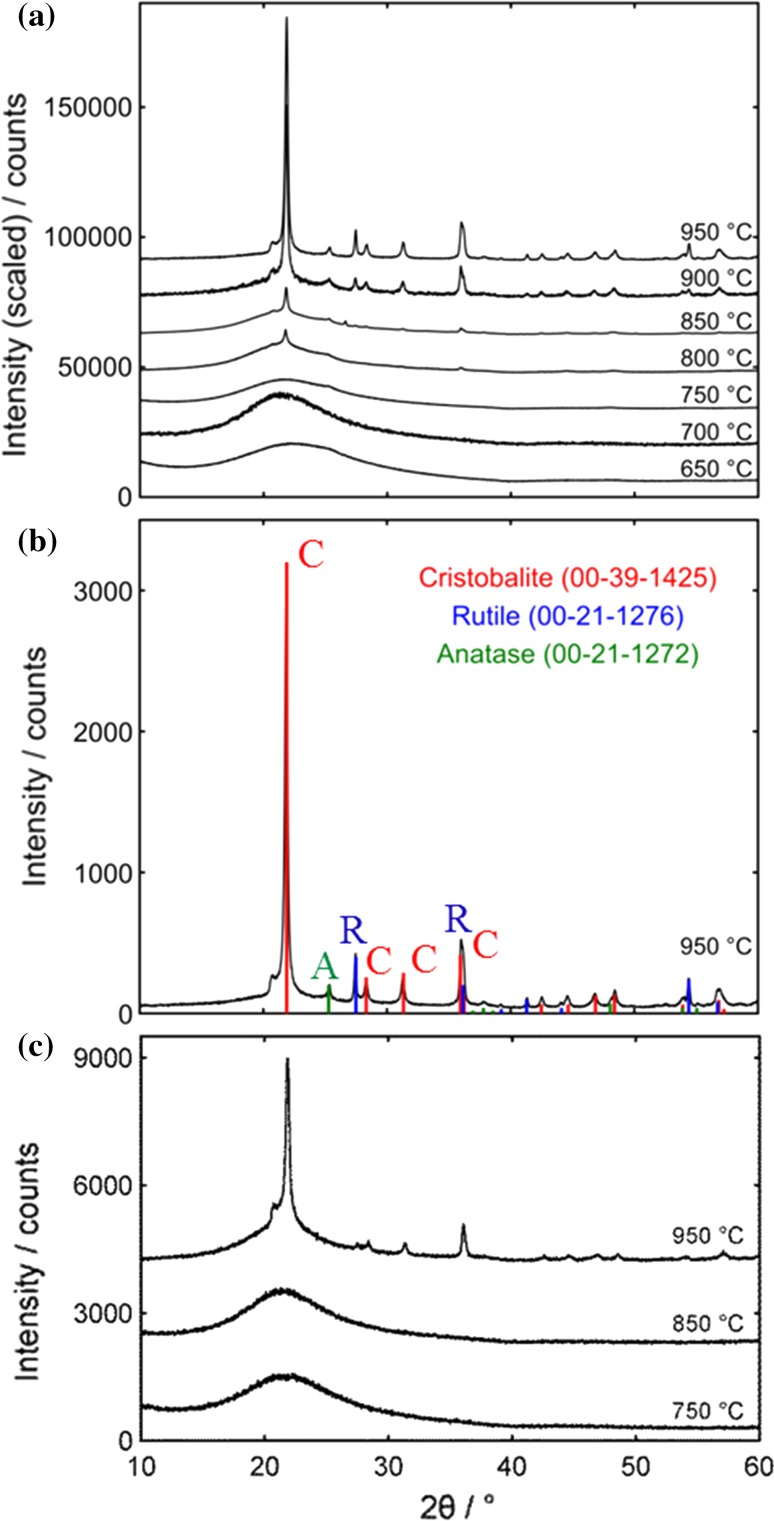
PXRD data for titanosilicate and silica samples calcined at different temperatures. **a** Propagation of crystal growth in titanosilicates MTSM-650 to -950. **b** Titanosilicate MTSM-950. Sample appears completely crystallized with crystalline phases of cristobalite (C), rutile (R) and anatase (A). The predominant crystalline phase is cristobalite. **c** Propagation of crystal growth in silica samples S-750, S-850 and S-950

Since sample MTSM-750-3 displayed the most prominent presence of crystals via TEM, it was chosen as a base sample for further experiments and analysis. In order to identify the crystalline component, it was necessary to increase their amount so that characteristic PXRD peaks could be observed and used for phase identification. In this regard, MTSM-750-3 was synthesized several times and calcined at various temperatures from 650 to 950 °C, with 50 °C increments (Fig. 2a). Since the previous catalytic experiments were conducted from samples calcined at 750 °C, temperatures higher than that, i.e., 800, 850, 900 and 950 °C, were used to induce formation of more crystalline materials. Lower temperatures of 650 and 700 °C were also used to determine the threshold of crystallite formation. At these lower temperatures and at 750 °C, only a broad peak, characteristic of the amorphous titanosilicate was observed, similar to previous observations. A temperature of 800 °C was necessary to form a sufficient amount of crystals that can be identified via PXRD. A clear increase in crystal formation was observed with increasing calcination temperatures. At 950 °C, the titanosilicate appeared to be completely crystallized with no indication of the amorphous peak (Fig. 2b).

Three different crystalline phases were identified via PXRD, as cristobalite, rutile and anatase. Among these, the predominant phase was clearly observed to be cristobalite, which is a crystalline form of silica. The other two phases; rutile and anatase, which are crystalline forms of titania, appear to be present in only small quantities. This observation reveals a couple of surprising factors with respect to phase transition in titanosilicates: First, formation of cristobalite at a temperature as low as 800 °C is highly unusual, without the addition of alkali metal salts, and secondly, cristobalite is the predominant form of crystalline material formed, even though rutile and anatase can readily form at much lower temperatures. This led to the hypothesis that some other factor could be inducing the formation of cristobalite, which was previously unknown.

In order to verify whether this effect is induced by the presence of Ti^4+^, silica samples were synthesized according to procedures similar to that of the titanosilicates, with the exception of the addition of Ti(IV) *n*-butoxide. This was to ensure that all other factors related to the synthesis were kept identical, except for the presence of Ti^4+^ in the silica framework. The silica samples thus formed were calcined at 750, 850 and 950 °C. Unlike in the titanosilicate samples, there was no indication of crystal formation in silica until 950 °C (Fig. 2c). The predominant crystalline phase formed here was once again identified as cristobalite. Hence, in formation of cristobalite, a 150 °C difference exists between the titanosilicate and silica samples, indicating that the presence of Ti^4+^ may have an implication in its formation.

As stated previously, cristobalite is usually formed at 1470–1705 °C, in pure silica, and its occurrence at 950 °C is rare, especially without the addition of alkali metals. However, it has been reported that presence of quartz crystals can significantly lower the phase transition temperature of cristobalite formation [39]. It is possible that small amounts of quartz crystals were formed during the synthesis of the silica sample, and were undetected by XRD, which in turn induced the formation of cristobalite at 950 °C. Furthermore, cristobalite formation has been reported in silica sand, containing the quartz phase, calcined between 800 and 1000 °C [40], further indicating the impact of such heterogeneities on lowering phase transition temperatures, in silica.

**Figure 3:**
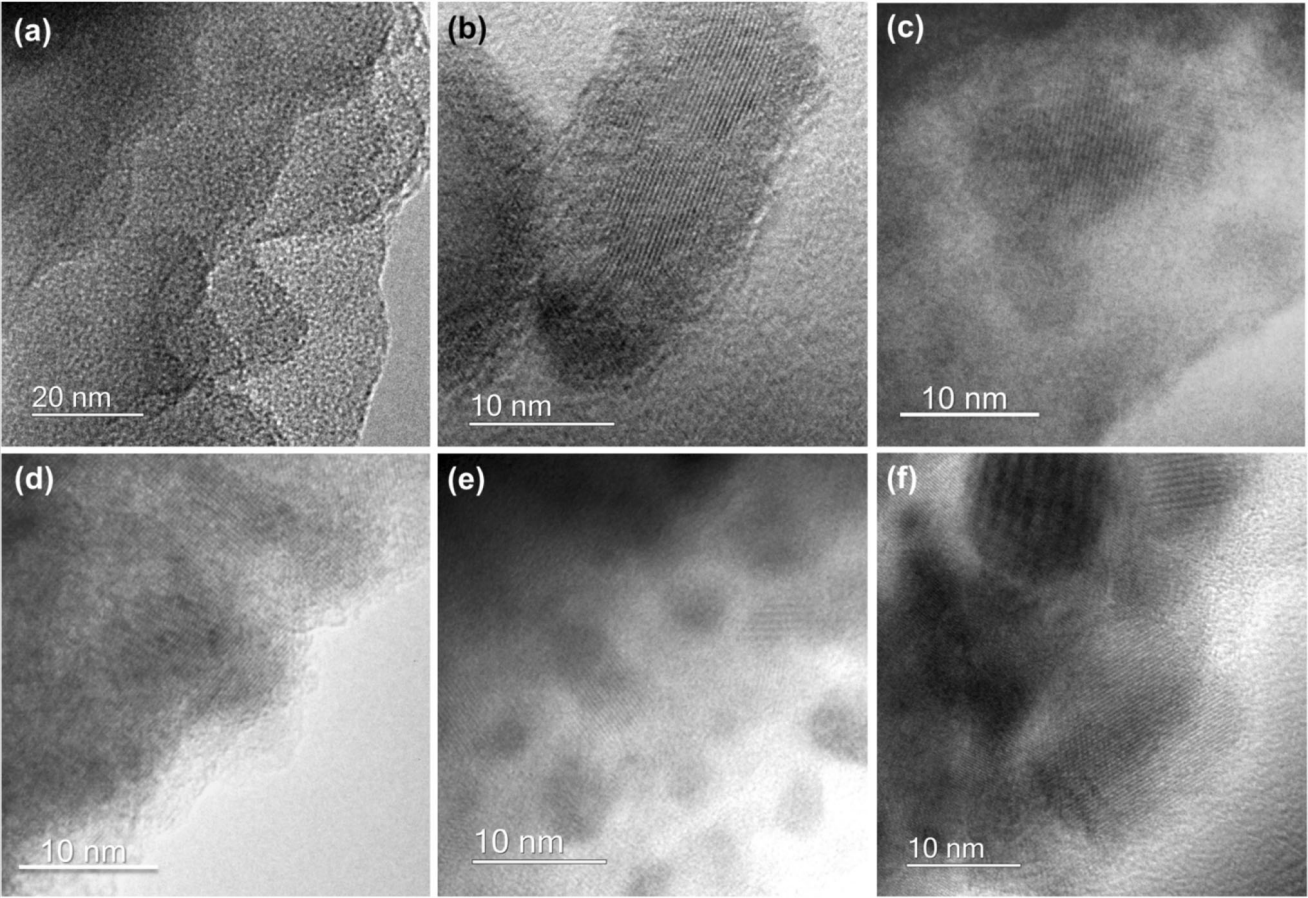
TEM images of titanosilicate MTSM-3 calcined at different temperatures showing formation of cristobalite. **a** MTSM-650, **b** MTSM-700, **c** MTSM-800, **d** MTSM-850, **e** MTSM-900 and **f** MTSM-950 °C. With the exception of (**a**), all other samples appear to show the presence of cristobalite

TEM imaging conducted on the samples revealed the presence of crystals in all titanosilicate samples, except for MTSM-650 (Fig. 3). This is indicative that temperatures of at least 700 °C are necessary for the formation of cristobalite, and that the crystals formed below 800 °C are present only in trace quantities and, therefore, cannot be detected via PXRD.

### The effect of cristobalite formation on titanosilicate catalysis

The fact that formation of cristobalite was confirmed via PXRD in titanosilicates at 800 °C, but only at 950 °C in the corresponding silica samples, is indicative that the phenomenon is induced by Ti^4+^. This is surprising, as the factors that normally facilitate the formation of cristobalite at lower temperatures, such as alkali metals, were not present during the synthesis. It is known that such secondary species, such as Na^+^, need to be present not just as impurities, but as components in significant amounts, in order for cristobalite formation to occur around 900 °C [19]. XPS studies confirmed that there was no presence of Na^+^ or other alkali metals in our materials and the only species present were O, Si and Ti, within Ti–O–Ti, Si–O–Si and Ti–O–Si environments (ESI, Table S3, Figure S6).

**Figure 4:**
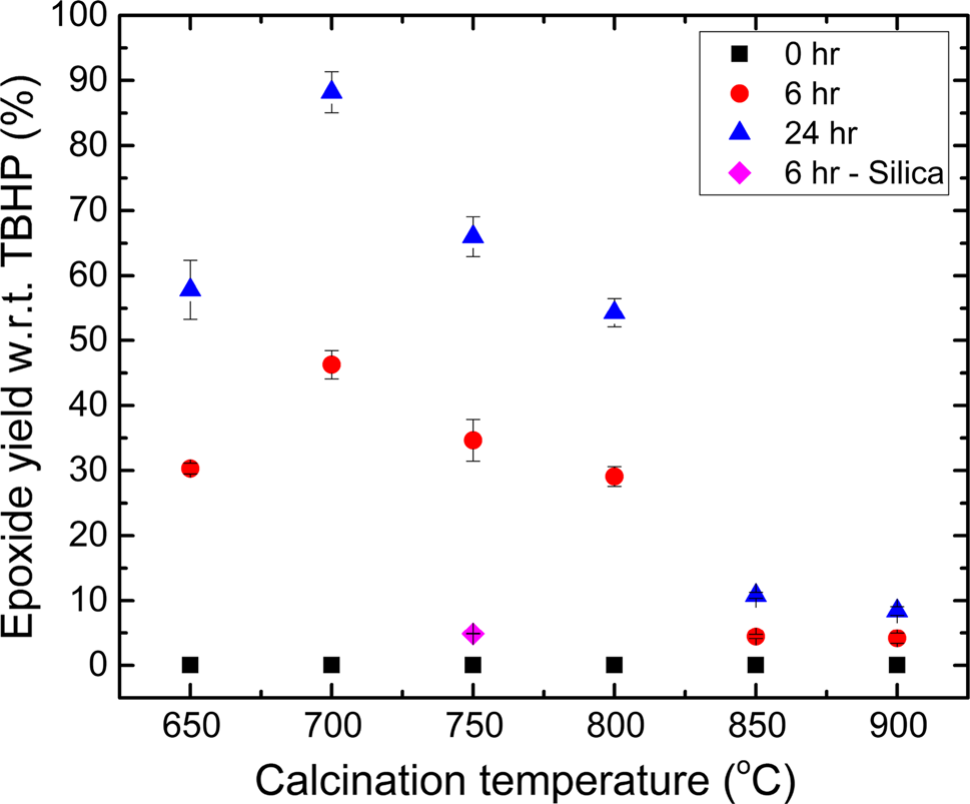
Catalytic results for titanosilicate samples MTSM-650 to -900 °C and silica sample S-750, in terms of epoxide yield with respect to TBHP

The next important question is whether the cristobalite formed had any impact on the catalytic activity of titanosilicates. In order to verify this, we conducted several catalytic experiments, where cyclohexene was oxidized using TBHP in decane, and titanosilicates MTSM-650, MTSM-700, MTSM-750, MTSM-800, MTSM-850 or MTSM-900 °C served as catalyst (Fig. 4). An equivalent silica sample S-750 was also used for comparison. Samples were taken out immediately after mixing in TBHP (which triggers the reaction), and designated as 0 h, followed by sampling at 6 and 24 h. The progress of the reaction was measured by the formation of cyclohexene oxide (i.e., epoxide of cyclohexene, which is the major product), with respect to TBHP (i.e., limiting reagent).

**Figure 5:**
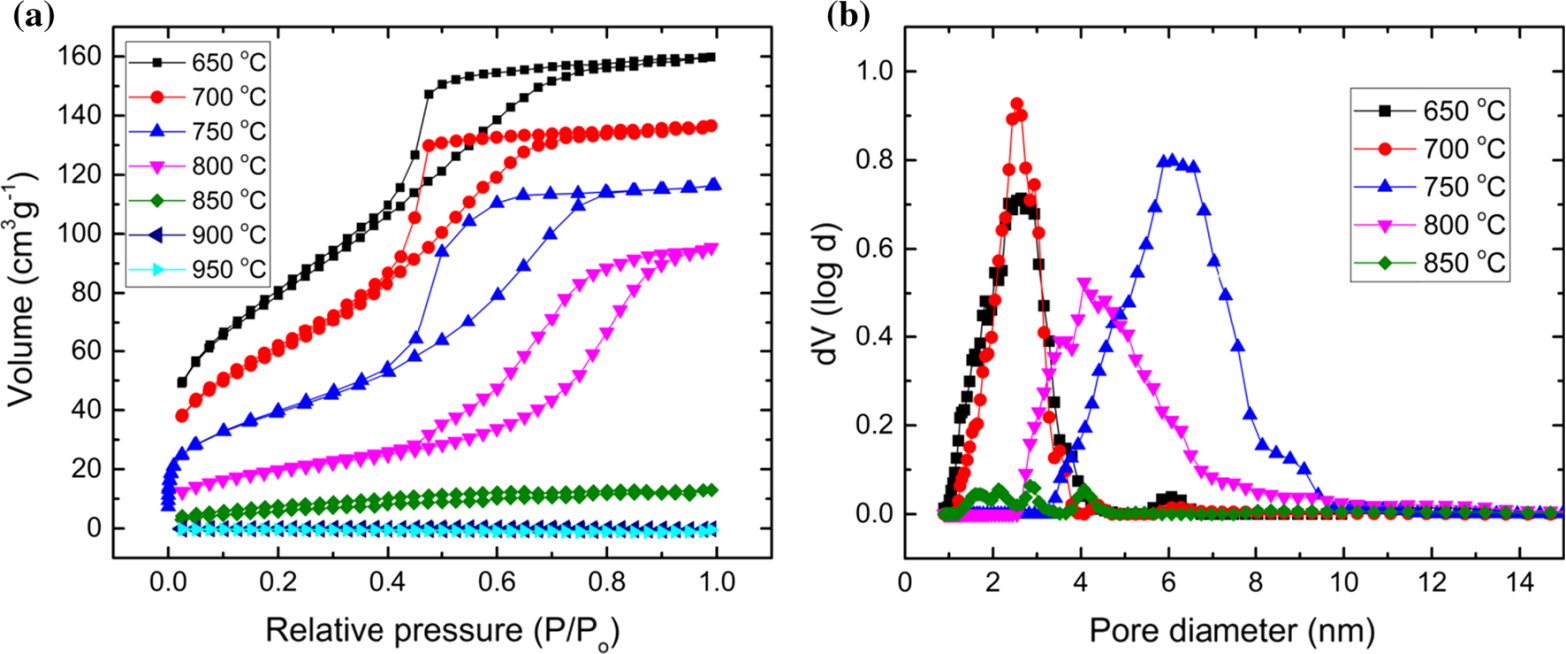
**a** Adsorption–desorption isotherms for titanosilicate samples MTSM-650, -700, -750, -800, -850, -900 and -950, analyzed using N_2_ at 77 K. **b** Pore size distributions for the above samples calculated using the NLDFT method

No significant product formation was observed immediately after mixing, at 0 h. After 6 h of reaction a trend became evident, where the highest epoxide yield was obtained by the catalyst MTSM-700, followed by similar yields within experimental error from the MTSM-750, -650 and -800 samples. Catalysts calcined at 850 °C and beyond had much lower epoxide yields. These observations can be explained by analyzing the nitrogen physisorption and XPS data of the samples. The BET surface area and the total pore volume decreased rapidly with increasing calcination temperature (Fig. 5a and Table 1). However, the amount of catalytically active tetrahedral Ti^4+^ sites, represented by the Ti 2p_1/2_ % from XPS analysis, did not follow this trend. The sample calcined at 700 °C, which had the second highest BET surface area, pore size and active sites %, was the best catalyst at 6 h, with 46% yield, surpassing the 650 °C sample (30% yield), which had the highest surface area and pore volume, and the 750 °C sample (34% yield), which had the highest % of active sites.

**Table 1:** Nitrogen physisorption and XPS data for MTSM-750-3 calcined at different temperatures

Calcination temperature of MTSM samples/°C	BET surface area/m^2^g^−1^	Total pore volume/ccg^−1^	Micropore volume/%	Average pore diameter/nm	Tetrahedral Ti 2*p*_1/2_/%
650	295	0.239	37	2.5	0.65
700	222	0.203	24	2.6	1.56
750	142	0.165	6	6	1.7
800	71	0.143	0	4.5	0.53
850	–	–	–	–	0.51
900	–	–	–	–	0.66
950	–	–	–	–	0.78

Despite having high surface area and pore volume, the MTSM-650 sample had a high percentage of micropores, which can act as bottlenecks that prevent the reagents from reaching the catalytic active sites. This leads to a reduction in overall catalytic activity and can be detected as a gating effect, which creates a gap between the adsorption–desorption BET isotherm branches, beyond the hysteresis loop (Fig. 5a). MTSM-650 also had less than half of the % of active sites of MTSM-750, further explaining its relatively low activity. On the other hand, MTSM-750, despite having a high % of active sites, had a much lower surface area and pore volume, and the combined effect was reflected in its lower catalytic activity, compared to MTSM-700. Thus, the sample with a combination of the most favorable features for catalysis appears to be the best catalyst, in line with our previous study [36]. Continuing this trend, MTSM-800 gave a low yield of 29%, and samples with higher calcination temperatures were shown to be even poorer catalysts. The pore network appeared to have collapsed in samples calcined at higher temperatures of 850, 900 and 950 °C, hindering the reagents from reaching the catalytic sites and, thus, leading to low catalytic activity.

We have previously studied the kinetics of this reaction and determined that ~ 24 h is sufficient for its completion under the above-mentioned reaction conditions, and hence, samples were also collected at this time. The trend in epoxide formation appeared to be similar after 24 h, as the sample calcined at 700 °C gave the best epoxide yield of 88%. This was followed by 750, 650 and 800 °C samples with yields of 66, 58 and 54%. It is noteworthy that the latter three yields are within each other’s experimental error (~ 17%) and are hence effectively the same. MTSM-850 and MTSM-900 samples gave rather low yields of 11% and 8%, respectively, in keeping with the previous trend. The activities of each sample once again can be linked with the pore structure and the % of active site, along with the amounts of cristobalite formed at each temperature.

It must be noted that an increasing amount of cristobalite is formed with increasing calcination temperature, according to the PXRD analysis. Even in low-temperature samples such as MTSM-650, -700 and -750, it is reasonable to assume that cristobalite is present in smaller quantities, as evidenced by TEM, despite the absence of characteristic peaks in PXRD. As evidenced by the BET data, formation of increased amounts of cristobalite crystals causes the pore network of the materials to collapse, resulting in progressively lower catalytic activity with increasing temperature. This was particularly evident for samples calcined at 850 °C and above. Therefore, MTSM-900 and -950 °C gave very low product yields, despite having similar % of active sites to MTSM-650. Hence, it can be concluded that an increased amount of cristobalite is detrimental to titanosilicate catalysis, as its formation leads to breakdown of the pore network. When present in smaller quantities, this effect is far less evident, and the catalytic activity is then dependent on established factors such as high surface area, pore volume and % of active sites of the titanosilicate. Thus, cristobalite formation appears to have a negative effect on the catalytic activity of titanosilicates.

### Significance of cristobalite formation in titanosilicates at lower temperatures

This study revealed three significant factors that are imperative to titanosilicate catalysis, particularly when the material synthesis requires a calcination step: First, formation of non-active crystalline phases, such as cristobalite, can occur at much lower temperatures than previously reported, without the addition of any alkali metals; second, Ti^4+^ can induce the formation of cristobalite; and third, the formation of significant amounts of cristobalite is detrimental to the catalytic activity of titanosilicates. These findings provide insight into how the process of calcination can have a significant impact on the quality control of the final product. Calcination is a step that is often overlooked during amorphous zeolite and titanosilicate production, as its purpose is to simply remove the hydrocarbon templating agents [17, 36]. In contrast, for crystalline zeolites and titanosilicates, calcination is a means to achieve the desired framework structure and may impact catalytic activity [41, 42]. Thus, for amorphous materials, it is important to select calcination temperatures that effectively remove the templating reagents, while not causing undesirable phase transformations. As demonstrated by this study, such processes can occur in titanosilicates, even under previously unknown conditions. When considering large-scale synthesis, the presence of undesirable phases, such as cristobalite, may contribute to significant losses in catalytic activity and cause loss of efficiency in the entire manufacturing process. Therefore, careful monitoring of calcination conditions and investigating the factors that may induce such anomalies are critical in developing efficient catalysts.

## Conclusions

We have demonstrated that cristobalite, a crystalline form of silica, can form in titanosilicates at relatively lower temperatures, without the presence of alkali metals, which are previously unknown conditions. PXRD studies revealed the presence of cristobalite crystals dispersed in an amorphous form of titanosilicate, MTSM, at 800 °C. However, HRTEM imaging revealed that these crystals can form in smaller quantities at temperatures as low as 700 °C. MTSM samples were synthesized according to previously known conditions and calcined at temperatures between 650 and 950 °C, in order to investigate the propagation of cristobalite formation. With annealing temperature, increased amounts of cristobalite were formed and the materials appeared to be completely crystallized at temperatures 900 °C and above. These titanosilicates were used to catalyze the epoxidation of cyclohexene with TBHP in order to investigate the effect of cristobalite formation on catalysis. The sample calcined at 700 °C (MTSM-700) was the most effective catalyst with 88% epoxide yield with respect to TBHP, after 24 h of reaction conducted at 60 °C, followed by MTSM-750, -650 and -800 samples. Materials calcined at higher temperatures were found to be extremely poor catalysts. The catalytic activity of these samples was influenced by their high surface area and mesopore volume, which favored the efficient transfer of reagents to the catalytically active sites. Increasing calcination temperatures allowed higher amounts of cristobalite crystals to be formed, which led to the collapse of the pore network and subsequent low catalytic activity. Investigation of such factors that lead to phase transitions in titanosilicates, under previously unknown conditions, will allow greater quality control in catalyst synthesis, via controlling calcination conditions.

## Electronic supplementary material

Below is the link to the electronic supplementary material.

Supplementary material 1 (DOCX 281 kb)

## Abbreviations

TBHP: *Tert*-butyl hydroperoxide
TEOS: Tetraethyl orthosilicate
DM: Doehlert matrix
PXRD: Powder X-ray diffraction
HRTEM: High resolution transmission electron microscopy
NLDFT: Non-local density functional theory
BET: Brunauer–Emmett–Teller
BJH: Barrett–Joyner–Halenda
EDX: Energy dispersive X-ray spectroscopy
XPS: X-ray photoelectron spectroscopy
