# Supplementary material for: Titanium(IV)-induced cristobalite formation in titanosilicates and its potential impact on catalysis
Source: J Mater Sci. 2018 Sep 6;54(1):335–45. doi: 10.1007/s10853-018-2869-0 (PMC6411274; doi:10.1007/s10853-018-2869-0)
Supplement: Supplementary file 1 — Supplementary material 1 (DOCX 281 kb) [file 10853_2018_2869_MOESM1_ESM.docx]

**Supporting Information**

Titanium(IV) Induced Cristobalite Formation in Titanosilicates and Its Potential Impact on Catalysis

Ayomi S. Perera,*^, 1^ Jeremy K. Cockcroft,^2^ Panagiotis Trogadas,^1^ Haiyue Yu,^1^ Nidhi Kapil,^1^ and Marc-Olivier Coppens*^,1^

^1^ Department of Chemical Engineering, Centre for Nature Inspired Engineering, University College London, Torrington Place, London, WC1E 7JE, UK, [ayomi.perera@ucl.ac.uk](mailto:ayomi.perera@ucl.ac.uk), [p.trogadas@ucl.ac.uk](mailto:p.trogadas@ucl.ac.uk), [haiyue.yu.15@ucl.ac.uk](mailto:haiyue.yu.15@ucl.ac.uk), [nidhi.kapil.15@ucl.ac.uk](mailto:nidhi.kapil.15@ucl.ac.uk), [m.coppens@ucl.ac.uk](mailto:m.coppens@ucl.ac.uk)

^2^ Department of Chemistry, University College London, 20 Gordon Street, WC1H 0AJ, UK, [j.k.cockcroft@ucl.ac.uk](mailto:j.k.cockcroft@ucl.ac.uk)

**1. Doehlert matrix optimization study:**[**^1^**](#_ENREF_1)

**Table S1.** Doehlert matrix experiments designed by changing two synthesis parameters: surfactant mass and homogenizing temperature.

| Sample | Factor 1 | Surfactant mass / g | Factor 2 | Temperature / ⁰C |
| --- | --- | --- | --- | --- |
| 1 | 0 | 7.9 | 0 | 80 |
| 2 | 1 | 9.4 | 0 | 80 |
| 3 | 0.5 | 8.6 | 0.866 | 97 |
| 4 | -1 | 6.4 | 0 | 80 |
| 5 | -0.5 | 7.2 | -0.866 | 63 |
| 6 | 0.5 | 8.6 | -0.866 | 63 |
| 7 | -0.5 | 7.2 | 0.866 | 97 |
| 8 | 1 | 9.4 | 0 | 97 |
| 9 | 0.5 | 9.0 | 0.866 | 104 |
| 10 | -1 | 7.9 | 0 | 97 |
| 11 | -0.5 | 8.3 | -0.866 | 90 |
| 12 | 0.5 | 9.0 | -0.866 | 90 |
| 13 | -0.5 | 8.3 | 0.866 | 104 |


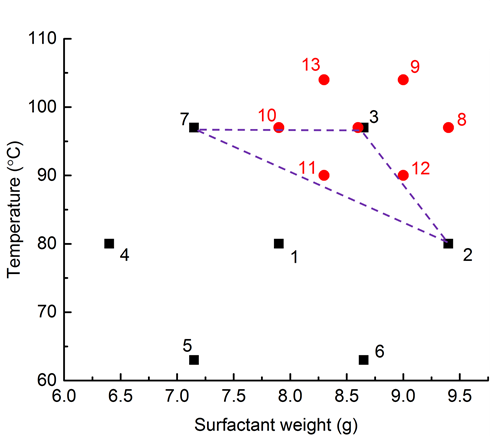


**Fig. S1.** Samples 1-13, corresponding to Doehlert matrices 1 (DM1 – samples 1-7) and 2 (DM2 – samples 8-13), represented in experimental design space. The triangle depicts the matrix region in DM1 that generated the three best catalyst samples.

Table S2. Results of cyclohexene epoxidation with TBHP using MTSM as catalyst after 24 h reaction in batch. (Refer to ESI, section 3 for details on calculations for a, b, c and d)

| Doehlert matrix number | Sample | Cyclohexene  conversion % ^a^ (±4) | TBHP  conversion % ^b^ (±8) | Epoxide yield  w.r.t TBHP % ^c^ | Selectivity % ^d^ |
| --- | --- | --- | --- | --- | --- |
|  | 1 | 7 | 83 | 56 | 65 |
|  | 2 | 12 | 94 | 53 | 55 |
|  | 3 | 17 | 97 | 66 | 68 |
| DM1 | 4 | 5 | 69 | 26 | 35 |
|  | 5 | 4 | 65 | 10 | 15 |
|  | 6 | 8 | 79 | 30 | 38 |
|  | 7 | 12 | 95 | 77 | 81 |
|  | 8 | 8 | 96 | 47 | 49 |
|  | 9 | 17 | 96 | 49 | 51 |
| DM2 | 10 | 15 | 95 | 54 | 57 |
|  | 11 | 10 | 97 | 49 | 50 |
|  | 12 | 13 | 93 | 48 | 52 |
|  | 13 | 11 | 97 | 54 | 56 |

**2. Reaction scheme, calibration curves and GC analysis:**

**
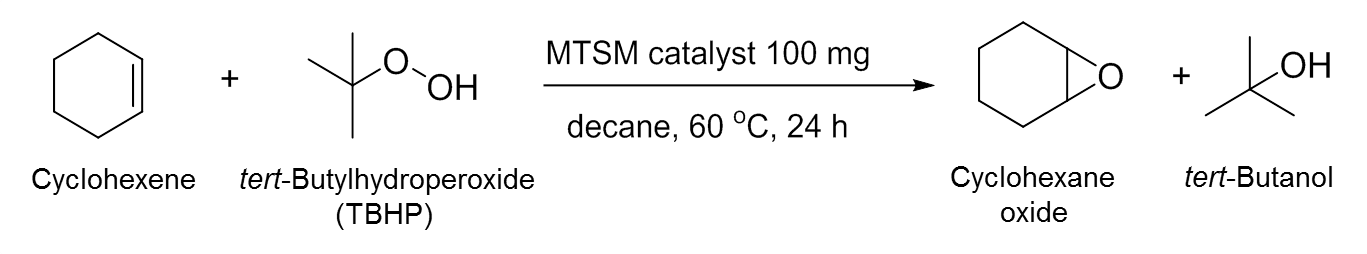
**

**Scheme S1.** Reaction of cyclohexene with TBHP under experimental conditions.

**Figure S2.** TBHP calibration curve.

**Figure S3.** Cyclohexene oxide calibration curve.

**Figure S4.** Cyclohexene calibration curve.


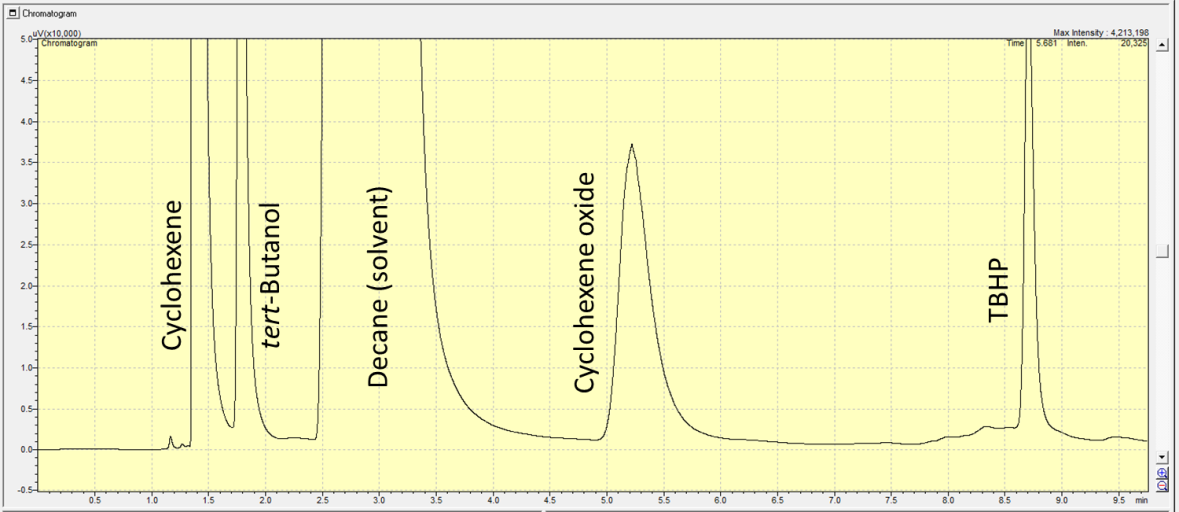


**Figure S5:** Gas chromatogram of reaction mixture, using DM1, sample 3 as catalyst, after 6 h of reaction.

**3. Details on calculations for catalytic experiments in Table S2**

Conversion of cyclohexene^a^ or TBHP^b^ (*x*):

$Conversion\left( x \right)=\left( 1- \left[ \frac{N_{x\left( t=t \right)}}{N_{x\left( t=0 \right)}} \right] \right) x 100\%$

*N_x_* = number of moles of *x*,

*t* = time

Yield of epoxide with respect to TBHP^c^:

$$Yield=\left[ \frac{N_{a\left( t=t \right)}}{N_{x\left( t=0 \right)}} \right] x 100\%$$

*N_a_* = number of moles of epoxide, *N_x_* = number of moles of TBHP

*t* = time

Epoxide selectivity (%)^d^:

Selectivity (%) = [moles of epoxide formed / total moles of TBHP reacted] x 100

**4. XPS analysis.**

Table S3. XPS data for MTSM materials and corresponding silica sample calcined at different temperatures.

| **Sample calcination temperature / ºC** | **Ti 2p _1/2_ / %** | **Ti 2p _3/2_ / %** | **Ti 2p total / %** | **Si 2p / %** | **O 1s / %** |
| --- | --- | --- | --- | --- | --- |
| 650 | 0.65 | 1.29 | 1.94 | 33.8 | 64.27 |
| 700 | 1.56 | 3.11 | 4.67 | 29.37 | 65.97 |
| 750 | 1.7 | 3.39 | 5.09 | 26.26 | 68.66 |
| 800 | 0.53 | 1.06 | 1.59 | 27.37 | 71.04 |
| 850 | 0.51 | 1.02 | 1.53 | 33.15 | 65.32 |
| 900 | 0.66 | 1.32 | 1.98 | 32.65 | 65.36 |
| 950 | 0.78 | 1.22 | 2 | 32.9 | 65.1 |
| Si only - 750 | 0 | 0 | 0 | 67.91 | 32.1 |

**5. EDX analysis**

Table S4. EDX analysis of MTSM materials calcined at different temperatures, depicting atomic %s of elements.

| **Sample calcination temperature / ºC** | **Ti / %** | **Si / %** | **O / %** |
| --- | --- | --- | --- |
| 650 | 2.38 | 34.45 | 63.17 |
| 700 | 3.35 | 34.48 | 62.16 |
| 750 | 3.69 | 34.51 | 61.81 |
| 800 | 2.56 | 34.39 | 63.05 |
| 850 | 2.32 | 30.85 | 66.83 |
| 900 | 2.36 | 28.09 | 69.54 |
| 950 | 2.10 | 29.71 | 68.18 |

References

1. A. S. Perera; P. Trogadas; M. M. Nigra; H. Yu; Coppens, M.-O., Optimization of mesoporous titanosilicate catalysts for alkene epoxidation via statistically guided synthesis. *J. Mater. Sci.* 2018, 53, 7279–7293.
